# Finite Element Analysis Modeling of a Novel Silicone Dressing

**DOI:** 10.7759/cureus.10629

**Published:** 2020-09-24

**Authors:** James Sieracki, Robert Wilkes, Eric R Bennett, Amy K McNulty

**Affiliations:** 1 Medical Solutions Division, 3M Company, St. Paul, USA; 2 Medical Solutions Division, 3M Company, San Antonio, USA

**Keywords:** silicone, foam, pressure injury, finite element analysis, strain

## Abstract

Introduction

In the United States (US), pressure injuries are believed to affect over 2.5 million people. The prevalence of pressure ulcers in the European Union (EU) is believed to be 13.7%. Recent guidelines have recommended the consideration of polyurethane foam dressings as part of pressure injury prevention strategies. This study assesses the reduction in tissue strain and stresses associated with the use of a new silicone foam dressing.

Methods

Finite element analysis (FEA) models were used to investigate the ability of silicone foam dressings to reduce tissue stress and strain energy density (SED) in the regions adjacent to the sacral bone. The loading modeled on the dressings was for combined compression and shear (modeling a patient lying in a 45° Fowler's position). Nine commercially available silicone foam dressings and a no-dressing control were modeled.

Results

FEA modeling showed that all silicone dressings tested, including Tegaderm™ Silicone Foam (TSF; 3M Health Care, St. Paul, MN) dressings, achieved reductions in tissue distortional stress and SED relative to no-dressing conditions. The use of silicone foam dressing results in a lower volume of tissue at higher stresses and deformation compared to no dressing.

Conclusion

The results presented indicate that TSF may provide an appropriate option for pressure ulcer prevention programs.

## Introduction

The incidence of pressure injury in the entire United States (US) healthcare settings was approximately 9.3% in 2015 [1]. An international pressure injury study found similar results with a pressure injury incidence of 2.4-12.0% [2]. A recent European systematic review found the overall prevalence of pressure ulcers to be 13.7%. Results ranged from 4.6-27.2% depending on the country [3]. In the US, pressure injuries are believed to affect over 2.5 million people at a cost of over $11 billion per year [4], and the overall incremental cost of treating a pressure injury has been estimated at $10,700 [5]. In Europe, treating a pressure ulcer has been shown to cost up to €470.49 per patient per day [6]. A recent retrospective study concluded that patients with pressure injuries experience statistically significant increases in length of stay, cost of care, and mortality as compared to patients without pressure injuries [7].

As its name implies, pressure is a major contributor to pressure injury, especially when the pressure occurs over a bony prominence. It is now recognized that shear stresses that lead to tissue deformation can exacerbate tissue injury as one layer of tissue moves relative to another [8]. Tissue shear is generated when friction exists between two surfaces that slide relative to each other. With respect to pressure injury, pressure, shear, and friction can lead to injury in the following manner: pressure over a bony prominence can compress and distort the skin and soft tissue, causing tensile and shear stresses [9]. The damage caused by pressure is dependent upon the amount of pressure and the time applied. It is generally believed that injury to muscle can occur in between one to three hours depending on the strain applied [10,11]. Shear stresses to tissue can also occur due to gravity. Any patient positioning angle between 0 and 90° will impart shear stresses to tissue due to the body’s tendency to slide down along the angled surface. Fowler's position (an angle of 45°) will cause a high combination of pressure and shear stresses in the sacral area [8]. Friction may exacerbate shear stresses in deeper tissue and muscle layers by keeping certain areas pinned against a surface while the body moves down the incline.

Pressure injury may start in deeper tissues, close to bony prominences, in response to sustained mechanical cellular deformation initiated by a localized load [9,11]. This localized load is often the result of body weight forces or external mechanical loading. Sustained deformation leads to inflammatory edema, resulting in increased interstitial pressures and even further cellular distortion. As cellular distortion increases, the integrity of the cell’s membrane and cytoskeleton are lost, resulting in cellular death. Unfortunately, with current sensors and experimental methods, it is only possible to measure external interface pressures at the skin surface interface. To understand internal pressures and deformation in deeper tissues generated from the mechanical load at the tissue interface, a computational simulation technique such as finite element analysis (FEA) is required [12]. Finite element models allow bioengineers to develop mathematical solutions to the complex and non-homogeneous tissue deformation (strains) and stress distributions that develop in response to external loading. The method breaks down large and complex problems into smaller parts termed finite elements. The equations that represent these elements are used to solve unknown values in the model. FEA models are an important tool that has been used to study pressure injury and technologies to reduce these injuries [13-16].

Recent guidelines have recommended the consideration of polyurethane foam dressings on heels and the sacrum as part of pressure injury prevention strategy [17]. FEA studies have shown that an anisotropic dressing (dressing with different material properties in different directions) can decrease the strain energy density (SED) under the sacrum by over 60% [18].

The current study uses FEA to assess the ability of a new five-layer anisotropic dressing to decrease internal pressures during supine or 45° Fowler's positioning in comparison to seven commercially available foam dressings as well as to the anisotropic dressing previously modeled. The FEA outcome measures modeled are peak stresses, SED, and percentage SED reduction.

## Materials and methods

This FEA modeling study was based on methods described by Levy and Gefen, 2017 [18], and was performed using FEBio Studio Software, a collaborative effort between the University of Utah's Musculoskeletal Research Laboratories and Columbia University's Musculoskeletal Biomechanics Laboratory [19]. FEA data extraction and histogram analysis utilized MATLAB (R2019b) (The MathWorks Inc., Natick, Massachusetts).

Computational models

Finite element models were developed and used to investigate the ability of foam dressings to reduce stress and shear on the skin and tissue surrounding the sacrum. The loading on the dressings was modeled with combined compression and shear (modeling a patient lying in a 45° Fowler's position). Nine commercially available silicone foam dressings were modeled as well as a no-dressing comparator.

Mechanical properties - dressings

The nine dressings studied in the current model were Tegaderm™ Silicone Foam Border Dressing (TSF; 3M Health Care, St. Paul, MN), Mepilex Border Sacrum Flex Dressing (MBF; Mölnlycke Health Care, Gothenburg, Sweden), Mepilex Border Sacrum Dressing (MBS; Mölnlycke Health Care), Aquacel Foam Adhesive Dressing (AFA; ConvaTec Inc., Greensboro, NC), Aquacel Foam Pro Dressing (AFP; ConvaTec Inc.), Biatain Silicone (BSD; Coloplast Corp., Minneapolis, MN), Optifoam Gentle Dressing (OGF; Medline Industries, Mundelein, IL), Allevyn Gentle Border Sacrum Dressing (AGB; Smith+Nephew, Hull, UK), and Allevyn Life (ALD; Smith+Nephew).

**Figure 1:**
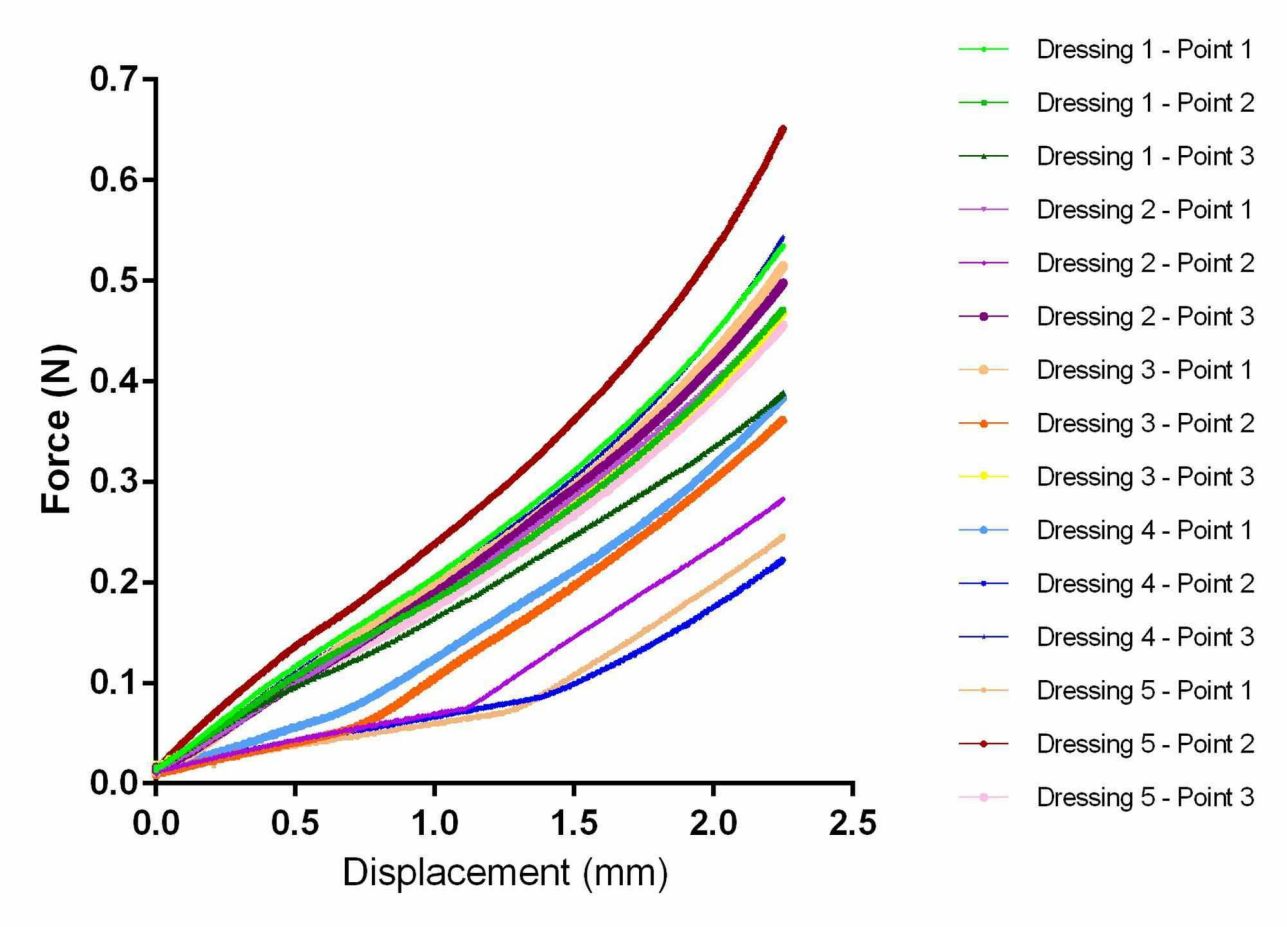
Load vs. displacement results for indentation testing of TSF dressings TSF: Tegaderm™ Silicone Foam

Each of these dressings comprised several layers. To simplify the model, the effective dressing modulus was obtained for each dressing by performing indentation testing. The testing equipment was an MTS Insight System™ (MTS Systems Corporation, Eden Prairie, MN). A 6-mm diameter cylinder was used to perform the indentation test at a rate of compression of 1 mm/sec. The probe peak displacement was set to half of the initial thickness for each dressing (50% compression). Three locations were probed per dressing, and five dressings were probed per dressing type. Representative output data from the testing for TSF can be found in Figure 1, and the corresponding effective dressing moduli (stiffness) computed from the data for all dressings can be found in Table 1. The constitutive model for the dressings was porous neo-Hookean using the modulus values from Table 1. A solid volume fraction of 2% was assumed.

Mechanical properties - physiology

**Figure 2:**
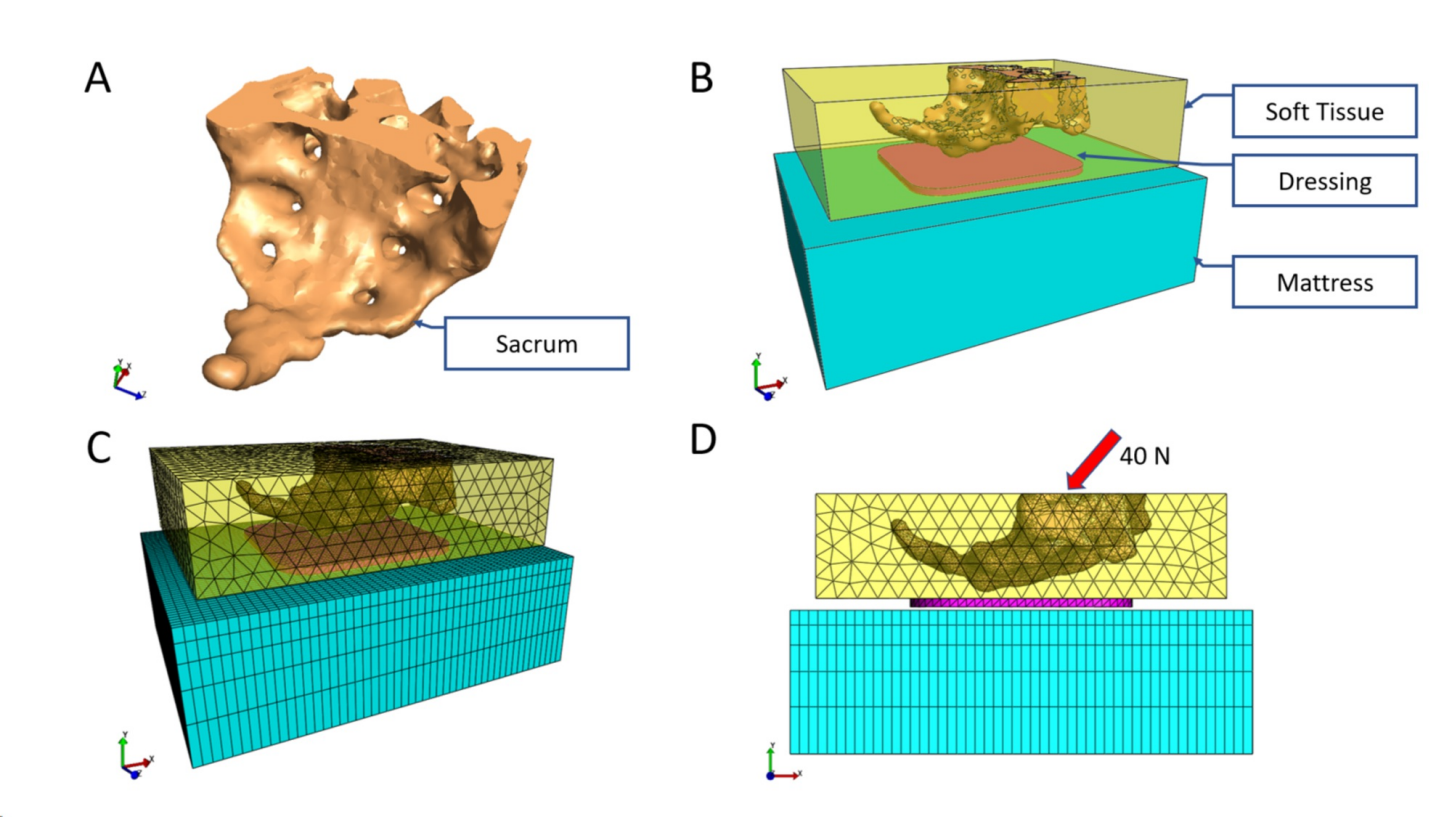
A) sacral bone 3D model; B) FEA model with sacral bone, soft tissue, dressing, and mattress; C) meshed FEA model; D) sagittal view indicating the arrangement of soft tissue, dressing, and mattress layers. Fowler's position loading of 40 Newtons is 45° from normal to the mattress FEA: finite element analysis

The bulk properties of human tissue and bone were studied, and their values published. The geometry of the sacral bone was obtained from a public domain site (https://www.turbosquid.com/3d-models/maya-igs-pelvis-solidworks/773673) (Figure 2A). The values used for the mechanical properties of the bone were previously published by Oomens et al.; Young’s modulus was 3.4 GPa and the Poisson’s ratio was 0.36 [20]. Soft tissue was modeled as a 200 mm x 250 mm x 75 mm block surrounding the sacral bone and foam dressing (Figure 2B-2D). The values used for the mechanical properties of the soft tissue were previously published by Oomens et al.; the shear modulus was 19.254 kPa, the bulk modulus was 1919.022 kPa, and the Poisson’s ratio was 0.495 [20]. The constitutive model for the soft tissue was incompressible neo-Hookean. The foam mattress was modeled as a block of a similar size to that of the soft tissue. The values used for the mechanical properties of the foam mattress were previously published; the elastic modulus was 50 kPa and the Poisson’s ratio was 0.3 [21-23].

Forces and loadings

For the Fowler's position FEA model, a displacement was applied to the sacral bone, 45° in the caudal-direction from perpendicular to the mattress (Figure 2D) until the total force returned on the mattress reached 40 N (Figure 2D) [21]. Dressings were placed in the ideal location, per manufacturers’ instructions for use. For each dressing modeled, the perimeter dimensions of the TSF Border Dressings were used (Figure 2B), and only the thickness and material parameters varied between dressings. The coefficient of friction between the foam mattress and the foam dressing was 0.4, representing a somewhat sticky interface. This value was used in Levy and Gefen, 2017, and was selected for use in the current paper to make results comparable [18]. In the current model, dressings adhered to the soft tissue with a no-slip relationship.

Biomechanical quantification

Given the three-dimensional nature of this model, quantitative outputs were summed elementwise for the soft-tissue volume. A volume-weighted SED histogram was generated for each dressing compared to the no-dressing case. The SED parameter considers the net effect of the tissue deformation and stresses in multiple directions and is representative of the total energy stored per unit volume of tissue at a given point in the tissue volume. For output, color contour plots (“heat maps”) were generated from the computational model, showing the magnitude and distribution of SED in the sagittal plane. Also, contour plots were generated to display the distortional (shear) stresses in the axial and sagittal planes.

Statistical methods

For the FEA model, SED histogram data were pooled from the soft tissue block into two categories: 0-0.5 kPa; 0.5 kPa and greater. Following this, percentage SED reduction was derived by the formula ((AUC(no dressing) - AUC(dressing))/AUC(no dressing))*100.

## Results

Mechanical properties - dressings

Due to the multilayer design of all dressings tested, sample-to-sample variability in the mechanical properties of the dressings was found. An example of this dressing variability is shown in Figure 1 for TSF where the force required for any displacement differed between points tested. Because of this, the FEA model was run conservatively using the lowest modulus value from each dressing tested (Table 1).

**Table 1:** Commercially available dressings’ model parameters and SED reduction results SED: strain energy density; AGB: Allevyn Gentle Border Sacrum Dressing; ALD: Allevyn Life Dressing; AFA: Aquacel Foam Adhesive Dressing; AFP: Aquacel Foam Pro Dressing; BSD: Biatain Silicone Dressing; MBF: Mepilex Border Sacrum Flex Dressing; MBS: Mepilex Border Sacrum Dressing; OGF: Optifoam Gentle Dressing; TSF: Tegaderm™ Silicone Foam Border Dressing

Dressing	Foam stiffness (kPa)	Foam thickness (mm)	% SED reduction in total	% SED reduction of <0.5 kPa	% SED reduction of ≥0.5 kPa
AGB	30.8	4.10	25.2	25.1	37.8
ALD	14.2	7.00	23.8	23.8	31.1
AFA	7.4	3.60	20.9	20.9	30.9
AFP	23.5	3.75	23.0	23.0	30.3
BSD	11.3	5.50	21.6	21.6	29.0
MBF	13.9	4.50	22.0	22.0	30.7
MBS	17.5	4.50	23.7	23.7	34.8
OGF	17.5	3.30	24.1	24.1	35.5
TSF	19.0	5.50	24.6	24.6	35.3

Strain energy density

**Figure 3:**
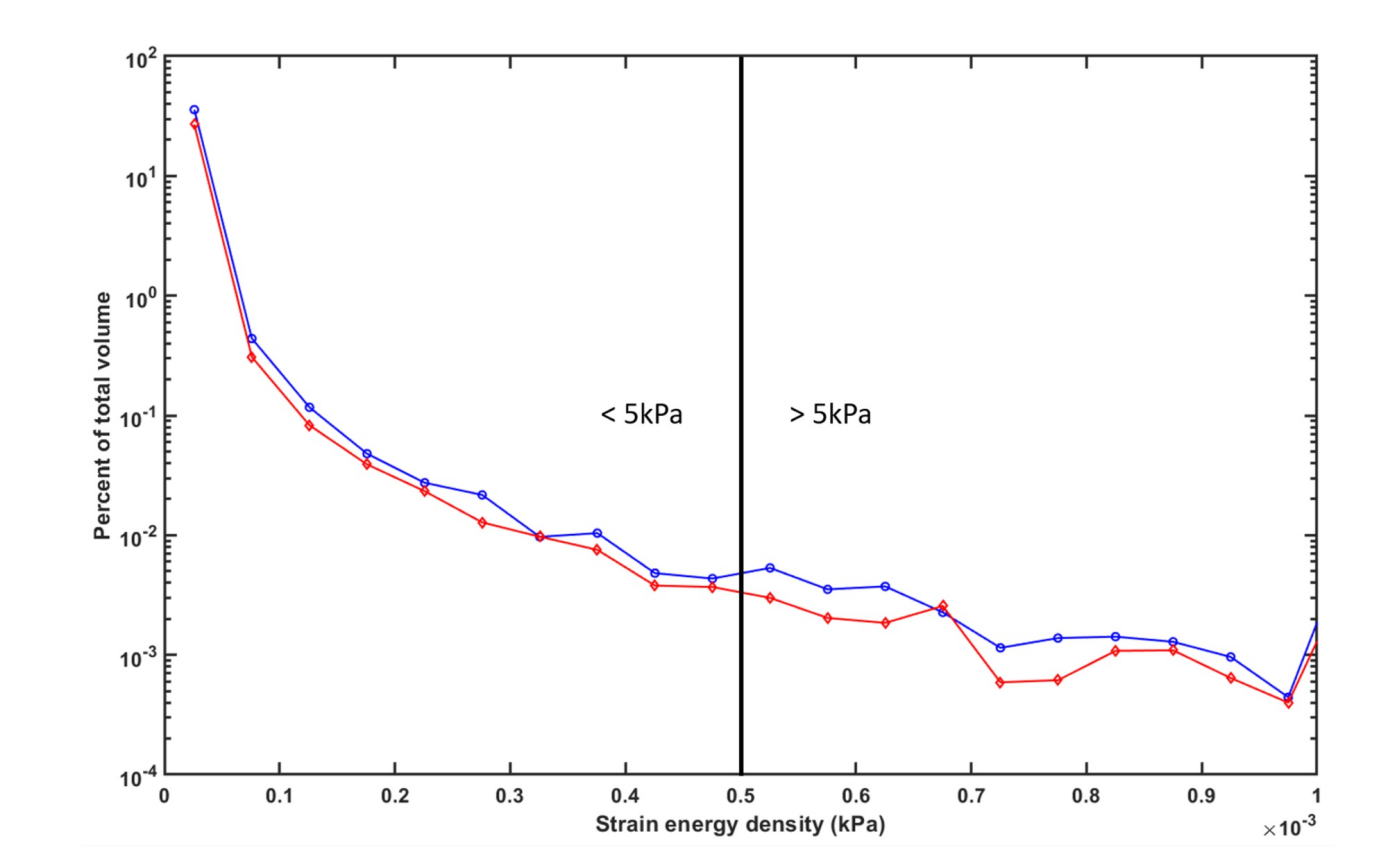
A representative semi-log plot of the strain energy density (SED) histogram for the modeling of soft tissue under combined compression and shear loads (Fowler's position) for no-dressing (blue line) and TSF dressing (red line) and at a friction of 0.4 between the dressing and mattress TSF: Tegaderm™ Silicone Foam

When compared to no dressing, it was found that the use of any of the foams tested greatly reduced the amount of tissue exposed to large deformation and stresses. The representative histogram plotted in Figure 3 for TSF shows the amount of tissue volume at specific SED values. The figure shows that TSF decreases the percentage of tissue volume at every SED tested between 0.05-1 kPa. All dressings tested (data not shown) exhibited a similar trend in that they decreased the volume of tissue exposed to deformation across the entire range of SED distribution analyzed (0.05-1.0 kPa) when loaded in combined compression and shear. The suite of dressings tested was able to substantially decrease the volume of tissue exposed to either lower SEDs (<0.5 kPa) by 20.9-25.1% or large deformations (SED of ≥0.5 kPa) by 29.0-37.8% when loaded in combined compression and shear (Table 1). The only dressing that did not exhibit at least 30% SED reduction of large deformations (SED of ≥0.5 kPa) was BSD (29% SED reduction). The dressing with the highest percentage of SED reduction for large deformations was AGB (37.8% SED reduction). TSF reduced the volume of tissue exposed to large deformations by 35.3%.

Stress concentration

**Figure 4:**
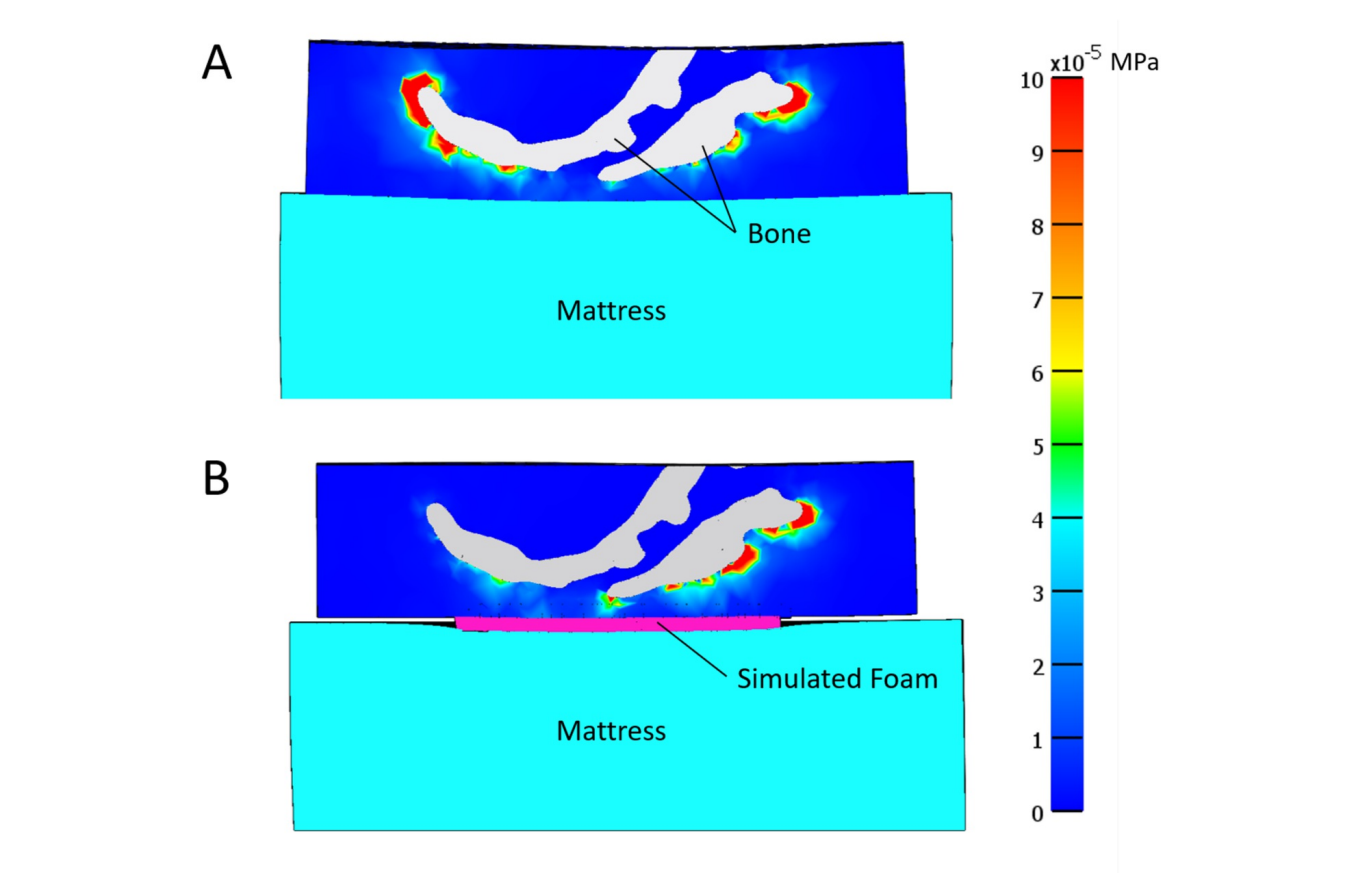
Representative strain energy density (MPa) contour plots in the sagittal plane of A) no dressing and B) TSF dressing cases under load TSF: Tegaderm™ Silicone Foam

**Figure 5:**
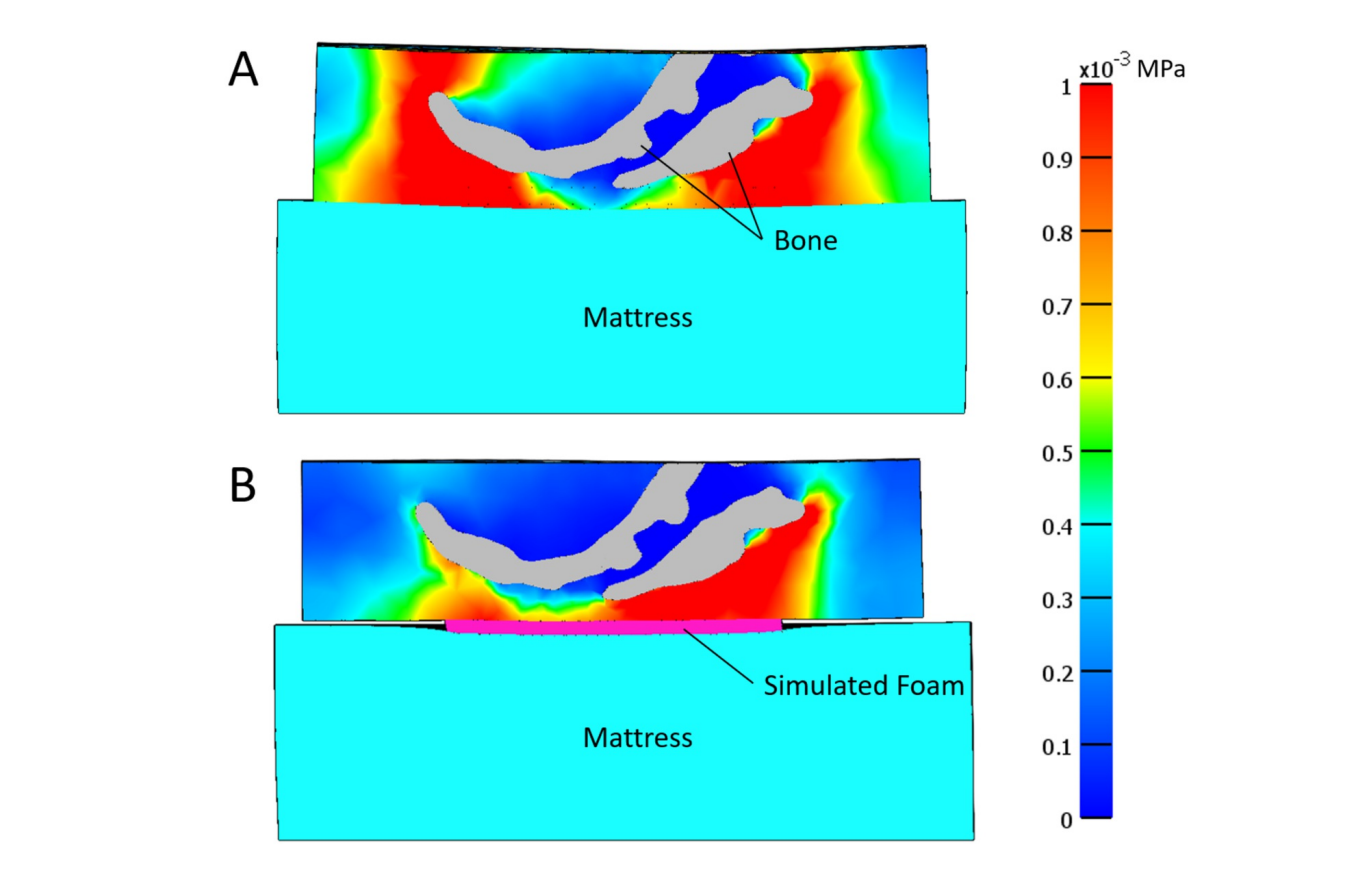
Shear stresses (MPa) in sagittal sacral cross-section with A) no dressing or B) with TSF dressing TSF: Tegaderm™ Silicone Foam

Figure 4A presents a strain energy density plot in the sagittal plane for the sacrum when no-dressing use was simulated. High shear/distortional stresses appear in regions of sharp bony prominences, i.e., at areas of tight radii (near the end of the sacrum). Figure 4B shows a representative plot of how dressing use (in this case TSF) reduced these stresses, especially around the tight radii as noted by the decrease in red areas (high MPa values) at the tip of the sacrum. All dressings tested showed a similar decrease in SED to TSF around the tip of the sacrum. Figure 5 is a representative figure showing the decrease in shear stress around the tip of the sacrum with simulated TSF use (Figure 5B) versus no dressing (Figure 5A). The decrease in shear stress around the sacrum is shown by the decrease in red (high MPa values). These trends were similar for all other dressings tested.

## Discussion

In this paper, computational models were used to calculate compression and shear forces exerted between the sacrum and mattress either with or without a prophylactic foam dressing. In a study to identify risk factors associated with pressure injury, immobility and a head-of-bed elevation greater than 30° were the two most frequently identified intrinsic factors [24]. For this reason, Fowler's position was modeled as a worst-case scenario in the present study. It is important to note that any bed position above 0° head elevation will lead to elevated shear levels. The typical patient position in a hospital bed is not supine and the current paper did not consider compression only as a relevant metric.

Modeling of prophylactic use of silicone foam dressings indicated that dressing use was associated with a decrease in high SED values (≥0.5 kPa) between 29.0-37.8% compared to the no-dressing equivalent (Table 1). The anisotropic dressings modeled herein have both a stiff and a compliant layer. This construction allows forces that would normally be concentrated around the patient’s bony prominences (i.e., sacrum) to be redistributed across the dressing. Previous work has also shown the benefit with respect to the strain energy reduction of a dressing that is constructed with both stiff and compliant layers as opposed to a dressing construction that is solely stiff [18].

Levy and Gefen have previously indicated that SED was a valuable measure for quantifying the potential for pressure injury [18]. SED distributions have correlated with tissue damage in a rodent model [25]. Ceelen et al.'s 2008 paper used FEA modeling to calculate internal strains that occurred during 2 h of rodent hindlimb loading [25]. The study showed that SEDs that did not exceed 0.35 kPa correlated with damage in approximately 50-60% of the experiments. However, tissue SEDs of 0.45 kPa or above correlated to at least some damage in 100% of the experiments. Therefore, the authors suggested that a SED value of approximately 0.45 kPa can discriminate between damage and no damage. We chose a value of 0.5 kPa in the current paper to indicate a potential damage threshold. All dressings modeled effectively decreased tissue volumes exposed to these potentially damaging, high SED values. The TSF dressing was able to decrease tissue volumes exposed to high SED values of ≥0.5 kPa by 35.3% when compared to no dressing. Similar to what was observed by Levy and Gefen [18] for MBF, the TSF dressing was able to reduce shear stresses around tight junctions and directly under the sacrum.

Limitations

While this study has revealed important biomechanical mechanisms at play and is relevant in the context of the use of prophylactic dressings, it is important to discuss some of the limitations in the modeling approach. Firstly, as with all FEA tissue models, homogeneity of the tissue was assumed whereas actual tissue had multiscale variations in these properties. Secondly, the attachment between internal tissue and bone was assumed to be a perfectly bonded interface, while in reality, this is probably not the case in that the muscle fascia and the periosteal membrane allow for more relative motion than what was included in the model. The net result of this is likely that our stresses are overpredicted to some extent. Overall, these two limitations induce some uncertainties in the values of the outcome measures (stress and SED) predicted in an absolute sense. However, the relative comparisons between the treatment conditions (i.e., no dressings, vs. MBF, vs. TSF) are still valuable and the results provide important insights. From the perspective of the dressing, we chose to homogenize the multiple dressing layers into one layer with equivalent properties in compression and shear. This allowed for simplification of the model while still retaining the net interfacial normal and shear conditions at the tissue-dressing, and bed foam-dressing interfaces. Finally, the current study is based on a single-subject MRI data set for the sacral geometry and tissue properties from Oomens et al. [20], representing an average value for skin and fat combined. This introduces limitations in terms of understanding the effects of patient-to-patient variability as well as the effects of chronic disease on tissue properties. Future studies should consider sensitivity analysis around the effects of these varying parameters.

## Conclusions

The FEA modeling results in this study indicated that the silicone foam dressings tested may greatly reduce exposure to high levels of tissue strains under the sacrum. FEA modeling showed that all silicone dressings tested, including TSF, achieved reductions in tissue distortional stress and SED relative to no-dressing conditions. The use of silicone foam dressing results in a lower volume of tissue at higher stresses and deformation compared to no dressing. The strain reduction characteristics of the TSF dressing may help to prophylactically protect patients from pressure injury, especially when lying in a hospital bed in Fowler's position.

## References

[1] Kayser SA, VanGilder CA, Ayello EA, Lachenbruch C (2018). Prevalence and analysis of medical device-related pressure injuries: results from the International Pressure Ulcer Prevalence Survey. Adv Skin Wound Care.

[2] VanGilder C, Lachenbruch C, Algrim-Boyle C, Meyer S (2017). The International Pressure Ulcer Prevalence Survey: 2006-2015: a 10-year pressure injury prevalence and demographic trend analysis by care setting. J Wound Ost Cont Nurs.

[3] Moore Z, Avsar P, Conaty L, Moore DH, Patton D, O'Connor T (2019). The prevalence of pressure ulcers in Europe, what does the European data tell us: a systematic review. J Wound Care.

[4] Sen CK, Gordillo GM, Roy S (2009). Human skin wounds: a major and snowballing threat to public health and the economy. Wound Repair Regen.

[5] Leaf Healthcare (2020). Leaf Healthcare: the financial impact of pressure ulcers. California.

[6] Demarré L, Verhaeghe S, Annemans L, Van Hecke A, Grypdonck M, Beeckman D (2015). The cost of pressure ulcer prevention and treatment in hospitals and nursing homes in Flanders: a cost-of-illness study. Int J Nurs Stud.

[7] Bauer K, Rock K, Nazzal M, Jones O, Qu W (2016). Pressure ulcers in the United States' inpatient population from 2008 to 2012: results of a retrospective nationwide study. Ostomy Wound Manage.

[8] Orsted HL, Ohura T, Harding K (2020). Pressure, shear, friction and microclimate in context. International Review: Pressure Ulcer Prevention: Pressure, Shear, Friction And Microclimate. A Consensus Document.

[9] Takahashi M, Black J, Dealey C, Gefen A (2010). Pressure in context. International Review: Pressure Ulcer Prevention: Pressure, Shear, Friction And Microclimate. A Consensus Document.

[10] Gefen A, van Nierop B, Bader DL, Oomens CW (2008). Strain-time cell-death threshold for skeletal muscle in a tissue-engineered model system for deep tissue injury. J Biomech.

[11] Stekelenburg A, Strijkers GJ, Parusel H, Bader DL, Nicolay K, Oomens CW (2007). Role of ischemia and deformation in the onset of compression-induced deep tissue injury: MRI-based studies in a rat model. J Appl Physiol (1985).

[12] Bucki M, Luboz V, Lobos C, Vuillerme N, Cannard F, Diot B, Payan Y (2012). Patient-specific finite element model of the buttocks for pressure ulcer prevention--linear versus non-linear modelling. Comput Methods Biomech Biomed Engin.

[13] Linder-Ganz E, Shabshin N, Gefen A (2009). Patient-specific modeling of deep tissue injury biomechanics in an unconscious patient who developed myonecrosis after prolonged lying. J Tissue Viability.

[14] Xiao DZ, Wu SY, Mak AF (2014). Accumulation of loading damage and unloading reperfusion injury--modeling of the propagation of deep tissue ulcers. J Biomech.

[15] Verver MM, van Hoof J, Oomens CW, Wismans JS, Baaijens FP (2004). A finite element model of the human buttocks for prediction of seat pressure distributions. Comput Methods Biomech Biomed Engin.

[16] Oomens CW, Bressers OF, Bosboom EM, Bouten CV, Blader DL (2003). Can loaded interface characteristics influence strain distributions in muscle adjacent to bony prominences?. Comput Methods Biomech Biomed Engin.

[17] (2014). National Pressure Ulcer Advisory Panel, European Pressure Ulcer Advisory Panel and Pan Pacific Pressure Injury Alliance: Prevention and Treatment of Pressure Ulcers: Quick Reference Guide. http://www.epuap.org/wp-content/uploads/2016/10/quick-reference-guide-digital-npuap-epuap-pppia-jan2016.pdf.

[18] Levy A, Gefen A (2017). Assessment of the biomechanical effects of prophylactic sacral dressings on tissue loads: a computational modeling analysis. Ostomy Wound Manage.

[19] Maas SA, Ellis BJ, Ateshian GA, Weiss JA (2012). FEBio: finite elements for biomechanics. J Biomech Eng.

[20] Oomens CW, Zenhorst W, Broek M, Hemmes B, Poeze M, Brink PR, Bader DL (2013). A numerical study to analyse the risk for pressure ulcer development on a spine board. Clin Biomech (Bristol, Avon).

[21] Levy A, Frank MB, Gefen A (2015). The biomechanical efficacy of dressings in preventing heel ulcers. J Tissue Viability.

[22] Levy A, Gefen A (2016). Computer modeling studies to assess whether a prophylactic dressing reduces the risk for deep tissue injury in the heels of supine patients with diabetes. Ostomy Wound Manage.

[23] Sopher R, Nixon J, McGinnis E, Gefen A (2011). The influence of foot posture, support stiffness, heel pad loading and tissue mechanical properties on biomechanical factors associated with a risk of heel ulceration. J Mech Behav Biomed Mater.

[24] Cox J, Roche S, Murphy V (2018). Pressure injury risk factors in critical care patients: a descriptive analysis. Adv Skin Wound Care.

[25] Ceelen KK, Stekelenburg A, Loerakker S (2008). Compression-induced damage and internal tissue strains are related. J Biomech.

